# Microarray Analysis of HIV Resistant Female Sex Workers Reveal a Gene Expression Signature Pattern Reminiscent of a Lowered Immune Activation State

**DOI:** 10.1371/journal.pone.0030048

**Published:** 2012-01-26

**Authors:** Elijah M. Songok, Ma Luo, Ben Liang, Paul Mclaren, Nadine Kaefer, Winnie Apidi, Genevieve Boucher, Joshua Kimani, Charles Wachihi, Rafick Sekaly, Keith Fowke, Blake T. Ball, Francis A. Plummer

**Affiliations:** 1 Kenya Medical Research Institute, Nairobi, Kenya; 2 Department of Medical Microbiology, University of Manitoba, Winnipeg, Canada; 3 University of Montreal, Montreal, Canada; 4 Public Health Agency of Canada, Winnipeg, Canada; 5 University of Nairobi, Nairobi, Kenya; University of Cape Town, South Africa

## Abstract

To identify novel biomarkers for HIV-1 resistance, including pathways that may be critical in anti-HIV-1 vaccine design, we carried out a gene expression analysis on blood samples obtained from HIV-1 highly exposed seronegatives (HESN) from a commercial sex worker cohort in Nairobi and compared their profiles to HIV-1 negative controls. Whole blood samples were collected from 43 HIV-1 resistant sex workers and a similar number of controls. Total RNA was extracted and hybridized to the Affymetrix HUG 133 Plus 2.0 micro arrays (Affymetrix, Santa Clara CA). Output data was analysed through ArrayAssist software (Agilent, San Jose CA). More than 2,274 probe sets were differentially expressed in the HESN as compared to the control group (fold change ≥1.3; p value ≤0.0001, FDR <0.05). Unsupervised hierarchical clustering of the differentially expressed genes readily distinguished HESNs from controls. Pathway analysis through the KEGG signaling database revealed a majority of the impacted pathways (13 of 15, 87%) had genes that were significantly down regulated. The most down expressed pathways were glycolysis/gluconeogenesis, pentose phosphate, phosphatidyl inositol, natural killer cell cytotoxicity and T-cell receptor signaling. Ribosomal protein synthesis and tight junction genes were up regulated. We infer that the hallmark of HIV-1 resistance is down regulation of genes in key signaling pathways that HIV-1 depends on for infection.

## Introduction

The disease AIDS perhaps ranks as one of the most devastating scourges of mankind. Since it was identified in 1983 more than 30 million people have died and another 33 million currently live with the virus [1], [2]. The majority of HIV-1 infections occur in Sub-Saharan Africa, where in some countries prevalence rates of more than 40% have been documented among antenatal clinic attendees [3]. Despite advances made in antiretroviral therapy, only a third of those requiring treatment receive it and new infections far outstrip those on therapy [4]. Similar to other previous viral epidemics, prevention through vaccination shall be the best approach. However, the nature of the AIDS virus to integrate itself to the host genome and its ability to constantly mutate and shield immunogenic components that induce protective antibodies, continue to pose challenges for an effective vaccine. Though a recent prime boost vaccine strategy, RV 144, provided signals that a HIV-1 vaccine was possible, its efficacy was only a modest 30% [5].

The presence of individuals who are highly exposed to HIV-1 but do not get infected, provide hope for a better understanding of correlates for protection that may lead to a more effective vaccine strategy. Highly exposed seronegative (HESN) populations have been identified among intravenous drug users [6], children born to seropositive mothers [7], [8], discordant couples [9], [10], and commercial sex workers [11]–[13]. The findings in 1996 on the protective effect of the CCR5Δ32 allele in HIV-1 infection [14] shifted focus to host genetics as probable cause of HIV-1 resistance. Over the past 10 years, approximately 40 genes have been documented from HESN populations as possible candidates for differential host susceptibility to HIV-1 [15]. This include selected human leukocyte antigens, HLA [16], TRIM5α [17], specific KIR-HLA associations [18], and APOBEC3G [19]. However, only a fraction of HESN has the protective genotypes described in the literature. Previous gene discovery studies have focused on single gene approaches which may have missed other compelling gene candidates and overlook interactions between different genes or genetic pathways. Microarray expression profiling provides new opportunities for simultaneous analysis of thousands of messenger RNA expression patterns in tandem with their biological functions. Less data exist on the use of microarrays to identify biomarkers for HIV-1 susceptibility, including pathways that may be critical for successful anti-HIV-1 vaccine and therapeutic design. In a bid to determine biomarkers and genetic pathways that are unique to HESNs, we carried out a global whole blood gene expression profiling of HIV-1 highly exposed and uninfected individuals from a well-characterized female commercial sex worker cohort from Nairobi, Kenya.

## Results

### Gene expression outcome

**Figure 1:**
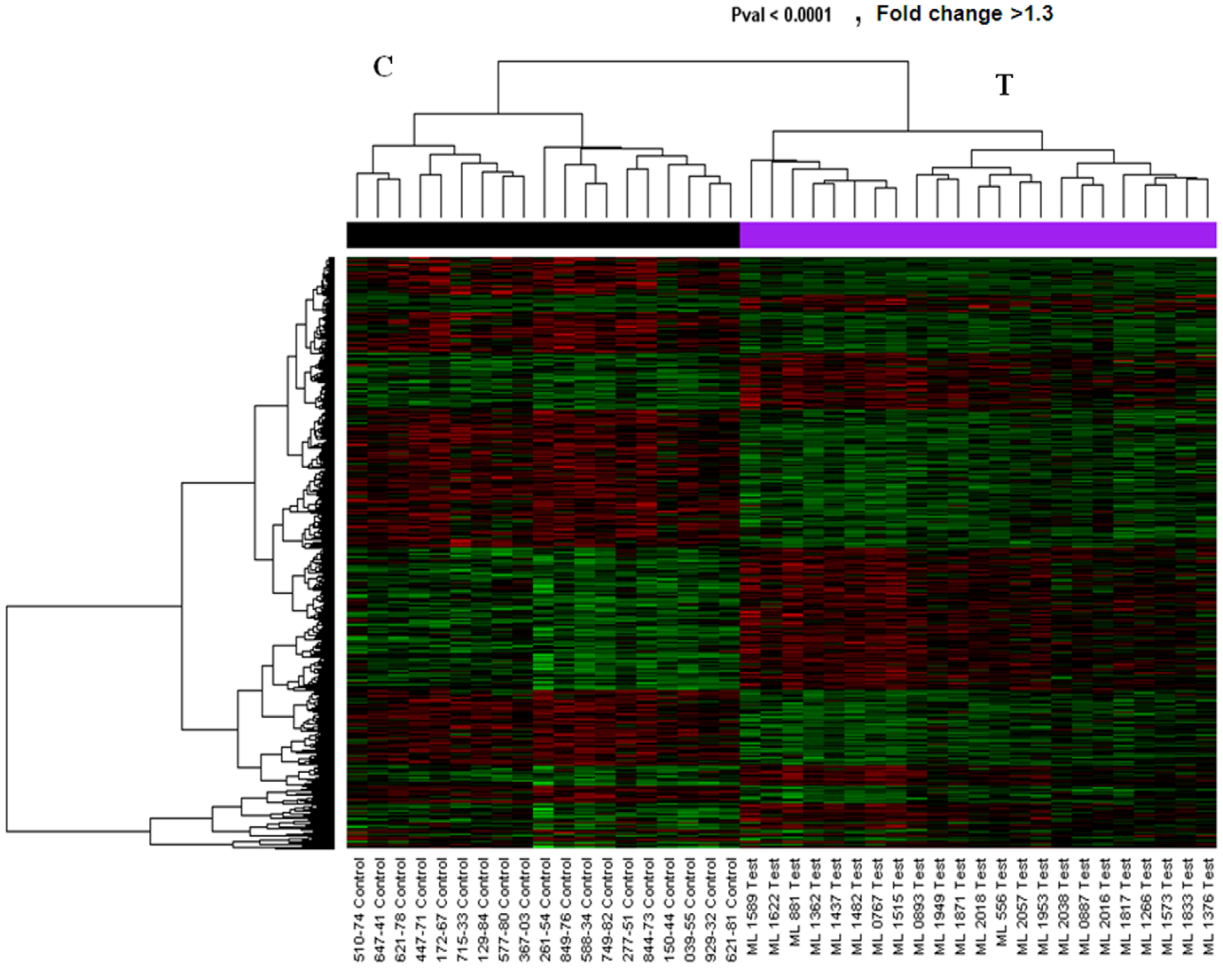
Heatmap of representative genes from a sample of HIV-1 highly exposed uninfected sex workers (Test) and susceptible HIV negative controls (control) evaluated through unsupervised hierarchical clustering algorithm. Each row on the Y axis represents a single gene probe and the phylograms represent distinct signaling pathways. The red color denotes up regulated genes while the green are down regulated.

Our primary objective was to investigate if there is a predictive gene expression pattern that defines the HESN phenotype. Gene expression data from the 86 micro arrays were normalized and all were included in the analysis. An unsupervised hierarchical clustering algorithm was run on the data to ascertain if the two populations could be separated into two distinct groups based on their gene expression profiles. Figure 1 show a distinct separation between the HESN (Test) and HIV negative controls (Controls).

**Figure 2:**
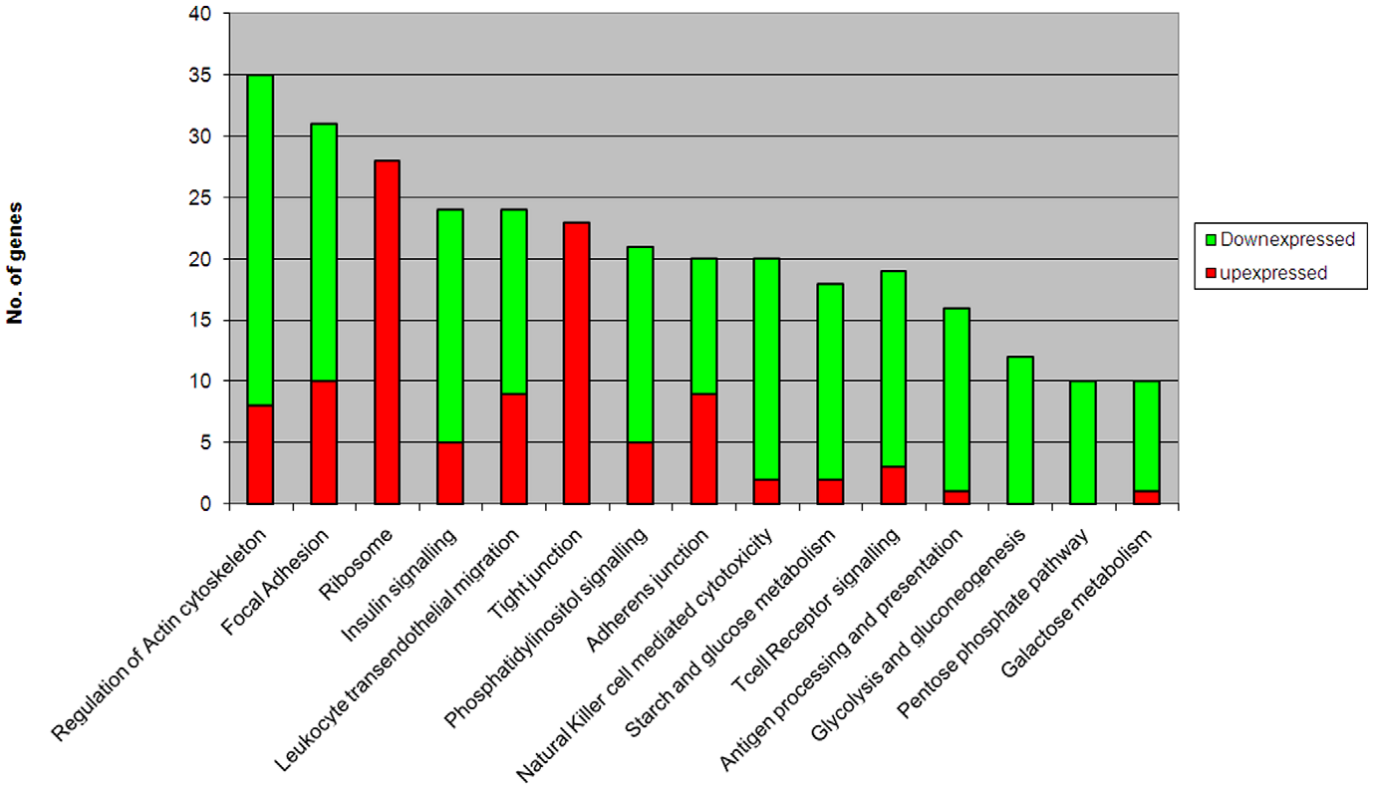
Proportion of differentially expressed genes in significantly affected pathways.

**Figure 3:**
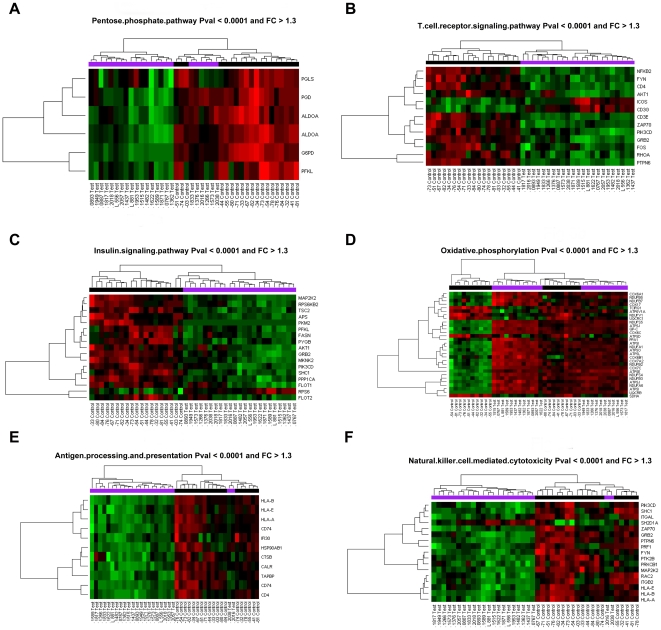
Heatmaps of the most differentially regulated signaling pathways among the HIV -1 highly exposed (Test) and HIV negatives (Control). Figure 3a -Pentose phosphate pathway; b) T cell receptor signaling; c) Insulin signaling, d) Oxidative phosphorylation, f) antigen processing and presentation, g) Natural Killer cell cytotoxicity. FC -fold change. Each row on the Y axis represents a single gene probe and the phylograms represent distinct signaling pathways. The red color denotes up regulated genes while the green are down regulated.

**Figure 4:**
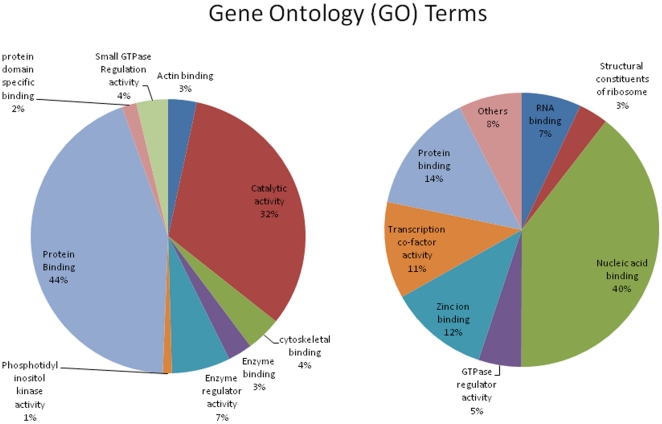
Gene ontology (GO) for downregulated genes (4a) and upregulated genes among the HIV-1 exposed uninfected sex workers.

Of the 54675 gene probe sets represented on the Affymetrix U133 plus 2.0 gene chip, 2,274 probe sets were differentially expressed in the HESN group as compared with the control group (fold change ≥1.3; p value ≤0.05) after correction with multiple testing using the Benjamin-Hochberg false discovery rate (FDR<0.05). Of the total differentially expressed transcripts, 462 (20%) could be mapped onto the KEGG signaling database. Figure 2 shows the 15 signaling events with the highest number of genes identified through the KEGG analysis. Majority of these pathways (13 of 15, 87%) were significantly down-regulated in the HESN group. The top ranked down-regulated pathways belonged to energy metabolism, cell adhesion, signal transduction and immune signaling categories. Glucose/gluconeogenesis and pentose phosphate phosphorylation (p<0.0003) were the most down-regulated metabolic pathways, together with the immune signaling events of natural killer cell cytotoxicity (p<0.008), antigen processing and presentation (p<0.0013) and T-cell receptor signaling. The key up-regulated pathways were the ribosome protein synthesis and tight junction. Figures 3 (a to g) show the heatmaps of representative genes in the significantly impacted pathways. Gene ontology (GO) classification of the differentially expressed transcripts identified protein binding as the major functional group among the down-regulated genes (Fig. 4a). In contrast, up-regulated genes were significantly associated with nuclear binding (Fig. 4b).

### Correlation with previous findings

More than 40% of genes that had earlier been reported to be associated with HIV-1 susceptibility in HESN populations were found to be differentially expressed in this dataset (Table 1). More significantly, genes that had been observed to be associated with the resistance phenotype in this cohort through single gene studies were confirmed to be differentially regulated. This included the Human leukocyte antigen (HLA) cluster (HLA-A, down-regulated, p value <0.001), Killer Immunoglobulin Receptors (KIR3DL1, down-regulated, *p* value <0.001) and Serpin B family (SerpinB13 up-regulated *p*<0.02). As noted in a previous study [20], expression of the CCR5 receptor cluster was not statistically different between the two populations (*p* value 0.6).

**Table 1:** Expression patterns of genes previously reported to be associated with HIV-1 Resistance.

Gene	Regulation	Fold Change	*p* value	Reference
APOBEC3G	up	1.1	0.7	[19]
CCL3L1	up	1.2	0.4	[65]
CCL4	up	1.1	0.6	[19]
CCL5	down	−1.1	0.6	[19]
CCR5	down	−1.1	0.6	[14]
CD207(Langerin)	up	1.2	0.4	[66]
CD209(DC-SIGN)	down	−1.2	0.02	[67]
CUL5	up	1.3	0.2	[68]
CXCL12(SDF-1)	down	−1.2	0.6	[69]
DEFA1	up	1.3	0.4	[71]
DEFA3	up	1.3	0.4	[71]
DEFB1	up	1.4	0.001	[15]
DEFB4	up	1.2	0.5	[15]
HLA-A	down	−1.1	0.01	[16]
HLA-B	down	−1.1	0.04	[16]
HLA-C	down	−1.1	0.03	[16]
IL4	down	−1.1	0.9	[70]
KIR2DL1	down	−1.9	0.02	[18]
KIR2DL2	down	−1.7	0.01	[18]
KIR2DL3	down	−1.8	0.07	[18]
KIR2DL4	down	−1.6	0.02	[18]
KIR2DL5	down	−1.6	0.02	[18]
KIR2DS1	down	−1.5	0.2	[18]
KIR2DS2	down	−1.8	0.1	[18]
KIR2DS3	down	−2.2	0.04	[18]
KIR2DS4	down	−1.6	0.1	[18]
KIR2DS5	down	−2.03	0.02	[18]
KIR3DL1	down	−1.9	0.01	[72]
KIR3DL2	down	−1.8	0.02	[72]
KIR3DL3	down	−1.8	0.08	[72]
PPIA(CyclophilinA)	up	1.5	0.002	[24]
TRIM5alpha	down	−1.6	0.1	[15], [17]
TSG101	down	−1.1	0.3	[15]
TLR2	down	−1.04	0.7	[73]
TLR4	down	−1.2	0.07	[73]
TLR8	down	−1.4	0.001	[73]

Table 2 shows the list of the top 50 genes identified as differentially expressed with a fold change of 2 and above. This is the first time many of these genes have been shown to be associated with a HESN population.

**Table 2:** Top 50 novel genes identified by microarray analysis with a fold change expression of 2 and above between the HESN and control group.

Affymetrix ID	Entrez	Gene Symbol	Chromosome	Regulation	*p*value
237953_at	1803	Dpp4	Chr2	up	1.99E-08
1569312_at	7705	ZNF146	Chr19	up	1.17E-07
237051_at	10463	SLC30A9	Chr4	up	1.27E-07
1559848_at	387338	NSUN4	Chr1	up	2.23E-05
241798_at	23244	SCC-112	Chr4	up	2.23E-05
244515_at	5713	PSMD7	Chr16	up	2.37E-05
243683_at	9643	MORF4L2	ChrX	up	2.91E-05
1557575_at	8987	GENX-3414	Chr4	up	3.09E-05
232279_at	23338	PHF15	Chr5	up	4.94E-05
233127_at	55422	ZNF331	Chr19	up	4.93E-05
225354_s_at	83699	SH3BGRL2	Chr6	down	5.32E-05
223683_at	84225	ZMYND15	Chr17	down	6.42E-05
212667_at	6678	SPARC	Chr5	down	6.57E-05
242191_at	200030	NBPF11	Chr1	up	6.60E-05
233302_at	64919	BCLIIB	Chr14	up	7.06E-05
206494_s_at	3674	ITGA2B	Chr17	down	1.01E-04
213258_at	7035	TFP1	Chr2	down	1.01E-04
221942_s_at	2982	GUCY1A3	Chr4	down	1.01E-04
201108_s_at	7057	THBS1	Chr15	down	1.41E-04
207114_at	80740	LY6G6C	Chr6	down	1.72E-04
227088_at	8654	PDE5A	Chr4	down	1.77E-04
1559126_at	23223	RRP12	Chr10	up	1.83E-04
216580_at	120872	RPL7	Chr12	up	2.08E-04
215859_at	56926	NCLN	Chr19	up	2.20E-04
240263_at	51106	TFB1M	Chr6	up	2.62E-04
227461_at	85439	STON2	Chr14	down	2.63E-04
201059_at	2017	CTTN	Chr11	down	2.69E-04
1560043_at	51706	CYB5R1	Chr1	up	2.79E-04
202729_s_at	4052	LTBP1	Chr2	down	3.04E-04
230014_at	51646	YPEL5	Chr2	up	3.18E-04
236841_at	374666	FAM39DP	Chr15	up	4.28E-04
239805_at	9058	SLC13A2	Chr17	down	4.75E-04
232570_s_at	80332	ADAM33	Chr20	up	6.13E-04
233087_at	64839	FBXL17	Chr5	up	6.13E-04
202275_at	2539	G6PD	ChrX	down	6.13E-04
229991_s_at	94121	SYTL4	ChrX	down	6.24E-04
230945_at	10120	ACTRIB	Chr2	up	6.80E-04
1558975_at	124402	FAM100A	Chr16	up	6.86E-04
1552750_at	117286	CIB3	Chr19	up	6.94E-04
209676_at	7035	TFP1	Chr2	down	7.28E-04
206254_at	1950	EGF1	Chr4	down	1.10E-04
226303_at	5239	PGM5	Chr9	down	1.33E-03
205524_s_at	1404	HAPLN1	Chr5	down	1.36E-03
222319_at	4676	NAP1L4	Chr11	up	1.41E-03
205409_at	2355	FOSL2	Chr2	down	1.46E-03
200785_s_at	4035	LRP1	Chr12	down	1.50E-03
212077_at	800	CALD1	Chr7	down	1.62E-03
219232_s_at	112399	EGLN3	Chr14	down	1.79E-03
1569542_at	166647	GP125	Chr4	up	1.90E-03
200661_at	5476	CTSA	Chr20	down	2.00E-03

### Quantitative RT-PCR validation

To validate the outcome of the microarray, representative down-regulated and up-regulated genes were picked for quantitative real time RT-PCR. Genes from one of the most down regulated signaling pathways- glucose\gluconeogenesis were randomly picked for qRTPCR analysis while a random sample of zinc finger proteins were selected to represent upregulated pathways. And to control for probable effect of sex work, samples from HIV seronegative sex workers (New Negatives) who had been involved in sex work for one year or less, were included in the qRTPCR analysis.

**Figure 5:**
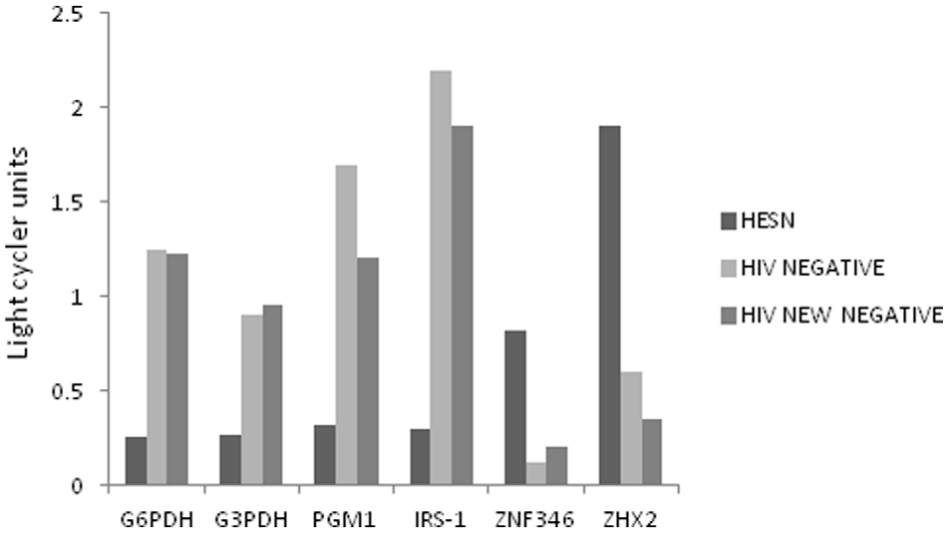
Outcome of a comparative quantitative RTPCR validation on randomly selected genes from the microarray analysis. Genes expression profiles from HESNs were compared with HIV uninfected non sex worker maternal child health clinic attendees (HIV negative) and HIV Negative new entrants into the sex trade (New Negatives). Each assay was a ratio gene quantity to its 18srRNA. G6PDH- glycose 6 phospate dehydrogenase; G3PDH-Glyceraldehyde 3 phosphate dehydrogenase; PGM-1-Phosphoglucomutase-1; IRS-1-Insulin receptor substrate-1; ZNF346-Zinc finger nuclease 346; ZHX2- Zinc finger and homeodomain protein 2.

The expression differences of the selected genes in HESN women as compared to the controls were similar to the microarray data (Fig. 5). No significant differences were observed in expression of the validated genes between the HIV seronegative sex workers and the HIV negative maternal-child health clinic attendees.

## Discussion

Our primary objective in this study was to investigate if there is a predictive gene expression signature pattern that defines the HESN phenotype in the Pumwani sex worker cohort. Apart from identifying candidate gene biomarkers, we used a systems biology approach to take into account the inherent complexity that may not be elucidated through single gene observations. We also used whole blood in an attempt to unravel the probable interplay between systemic intercellular networks that may be lost through individual cell type approaches. As controlling for HESN studies is often a challenge, we have attempted to include data from HIV negative new entrants into the sex worker trade as an additional control group to lessen confounding factors such as frequent exposures to other sexually transmitted infections. The overall outcome show a distinct difference in expression patterns between the HESN population and controls. Previous studies in this cohort have identified specific human leukocyte antigens (HLA) and interferon regulatory factors (IRF) as associated with resistance [16], [20], [21]. At the mucosal level, specific antibody responses as well as several antiproteases have been found to be elevated among the HIV-1 resistant women as compared to the controls [22], [23]. We noted a significantly differential expression pattern on the HLA types, confirming a probable role these markers may have on HIV-1 resistance. We also observed that some biomarkers, which had been identified in other HESN populations as possible contributors to the resistance phenotype, for example defensin β 1 [15] and certain polymorphs of cyclophilin A [24] were significantly up-regulated in our HESN group. These associations with previous findings reinforced our confidence in the platform we used.

The most up-regulated gene was the Dipeptidyl peptidase 4 (DPP4). The HESN group had a more than a 2 fold change over expression of this gene as compared to controls. DPP4, also known as CD26, is a 110 kDa protein that has been found to be one of the most ubiquitous soluble proteins. Its expression levels have been associated with cancer, diabetes and infectious disease [25]–[27]. The role of DPP4 in HIV-1 infection remains controversial. One of its known functions is to cleave dipeptides from the amino terminus of proteins containing a proline or alanine moiety at the ultimate position. A number of chemokines have this sequence at their termini. RANTES, a chemokine of the interleukin-8 superfamily, is a selective attractant for memory T lymphocytes and monocytes and has been shown to inhibit HIV infection by competitively binding CCR5 [28]. Proost *et al* [29] showed that truncated RANTES inhibited HIV-1 infection of mononuclear cells 5-fold more efficiently than intact RANTES. He confirmed that the truncation of RANTES was a result of the activity of DPP4 cleavage. Similarly, Morimoto and co-workers [30] have demonstrated that Jurkat T cell line expressing very high levels of DPP4 activity were resistant to HIV-1 infection. In contrast, several laboratories [31], [32] have reported a selective decrease in DPP4-expressing T cells in AIDS progression, suggesting that DPP4 rendered the cells more sensitive to HIV-1 infection. Our *in vivo* observations of clustering of high DPP4 expression among the HESN sex workers denote a possible HIV-1 protective effect.

In conformity with the high expression of DPP4, we observed a down-regulation of the insulin signaling pathway among the HESN women. Though we could not, in our studies, directly attribute the low expression of insulin genes to DPP4, the association of very high DPP4 with diabetes is a confirmed phenomenon [33], [34]. DPP4 inhibits the incretin hormones, glucagon-like peptide 1 and Gastric Inhibitory peptide, limiting release of insulin from the pancreatic cells and inducing a hyperglycemic state. Subsequently treatment protocols for type 2 diabetes have incorporated DPP4 inhibitors [35]. Though our follow up studies affirmed high DPP4 expression at the protein level [36], clinical evaluation of the HESN cohort did not confirm type 2 diabetes but an impaired fasting glucose state. This suggests that development of type 2 diabetes nay not be an automatic phenomenon in this cohort. We are carrying out further functional studies to determine the probable contribution and mechanism of DPP4 and insulin repression in the HESN phenotype.

The phospatidylinositol pathway was a key impacted signaling system among the HIV-1 resistant women (*p*<0.0001) This pathway consists of a cellular family of kinases that are activated by tyrosine phosphorylation and act as second messengers by regulating the phosphorylation of other kinases, including the ribosomal S6 kinases and the MAPK signaling network [37]. Because of its control of activation of many pathways, the inositol pathway is a critical mediator of various cellular processes. Down regulation of the phospatidylinositol system has been linked, to among others, low insulin levels. Insulin is a key regulator of the PI3K/AKT signaling system and insulin, through the insulin receptor substrate (IRS-1), provokes a rapid increase in levels of inositol phospholipids, PIP [38]. Additionally, PI3K was shown to be a key member of the pathway and was required for insulin induced glucose transport and glycogen synthesis. We postulate that the down expression of key immune pathways and cell adhesion molecule genes in our dataset may have been the result of the switch down of the PI3/AKT pathway, due in part by reduced insulin secretion. Furthermore evidence of direct effect of phosphotidylinositol signaling on HIV-1 infection has been noted. Francois *et al* [39], reported that inhibition of PI3K resulted in absence of HIV-1 infection of CD4+ T cells and macrophages with X4 and R5 pseudo typed viruses. Similarly Brown *et al* [40] has shown that blocking phosphotidylinositol phosphate (a substrate of P13K), with monoclonal antibodies inhibited HIV-1 infection by two HIV-1 primary isolates in human peripheral blood mononuclear cells. Chugh and colleagues [41] have demonstrated that blocking the PI3K/AKT pathway with Akt inhibitors dramatically reduced HIV-1 production from virus-infected macrophages. The implications of the observed down regulation of the phospatidylinositol pathway may hence be direct or through inhibition of HIV-1 dependency signaling events requiring PIP modulation.

In concordance with earlier findings from our group using specific cell subsets [42], we also observed lower basal T-cell gene expression, confirming a lowered immune activation state among the HESN group. Immune activation has been suggested to be the greater risk factor in HIV-1 infection and higher levels of activated T-cells in sub-Saharan African populations have been associated with higher HIV-1 prevalence [43]. This observation has been supported further by evidence of decrease in T-cell activation with successful antiretroviral use [44]. This low immune activation phenomenon, or immune quiescence, proposes a mechanistic profile of fewer activated cells presenting lesser targets to the AIDS virus and making it easier to clear infections that occur. Our findings of low basal gene expression in T-cells in our HESN population is also concordant with reports from similar populations that have shown lower levels of activated T-cells among seronegative partners of HIV-1 infected spouses [45]. Although these observations contrasts with reports from others [46]–[49], the gene expression patterns observed in our data bear a close metabolic signature pattern to that of quiescent T cells. Activated T cells have been noted to derive its ATP requirements from cytoplasmic glycolytic pathway [50], whereas quiescent T-cells derive metabolic needs through oxidative phosphorylation and other catabolic processes in the mitochondria [51], [52]. We noted that one of the few gene expression pathways that was up regulated by the HESNs was the mitochondrial oxidative phophorylation pathway (OXPHOS) with a concomitant profound down regulation of enzymes in the glycolysis pathway.

An up-regulation of DNA binding genes that have been associated with gene editing and silencing was a key phenomenon among the HESN. This included the Zinc finger (ZFN) and SMAD proteins encoding genes. Among the highly expressed ZFN genes were the Zinc Finger and Homeoboxes 2 (ZHX2) and Zinc Finger protein 20 (ZBTB20). ZHX2 has been identified as a transcription repressor gene [53] while ZBTB20 is a key repressor of alpha fetoprotein gene transcription [54]. SMAD3 is a strong enforcer of quiescence in T-lymphocytes through its antiproliferative and pro-survival signals [55]. In addition, the non-coding gene Xist, reported to trigger chromosome-wide gene repression [56], was highly up-regulated in the HESN women (*p* 2.80E^−10^).

In tandem with the overexpression of gene silencing factors at the transcriptional level, we observed dramatic down regulation of host factors reported to be important for successful HIV-1 infection. These factors included intergrins, a class of cell surface receptors that mediate linkages with the extracellular matrix and have been identified as HIV-1 receptors [57], catenins, components of the actin cytoskeleton shown to enhance HIV-1 infection by anchoring chemokine receptors CCR5 and CCR4 to the cell membrane [58], and NF- κB a known modulator of immune function that directly enhances HIV-1 transcription at the proviral level [59].

A previous in *vitro* genomewide screen identified more than 273 host genes that were essential for HIV-1 infection [60]. We noted that 66 of the genes identified in that study were among those differentially regulated by the HIV-1 resistant women at significant levels, most of which (61%) were down regulated (data not shown).

Our findings imply that a repertoire of genes acting in concert rather than a single determinant may be responsible for the observed HESN phenotypes in the Pumwani cohort. Our data also suggest that genes contributing to a lowered basal immune activation state, in tandem with a general repression of host HIV-1 dependency factors, may be a key contributor to the HIV-1 resistance phenomenon. Hence, the understanding of signaling and metabolic events that regulate immune activation may provide crucial information for the design of effective anti-HIV-1 preventive strategies.

## Materials and Methods

### Ethical Statement

This study was guided by the Helsinki Declaration on ethical principles for medical research involving human subjects. All studies were approved by University of Nairobi/Kenyatta National Hospital Ethical Review Board and the University of Manitoba Health Research Ethics Board. All patients provided written informed consent for collection of samples and subsequent analysis.

### Subjects

The Nairobi (Pumwani) commercial sex worker cohort was established in 1985 and has provided vital data that there might be biological mediated resistance to HIV-1 infection [11], [16], [20], [21]. Despite repeated exposures to HIV-1, a number of women in this cohort have remained HIV-1 uninfected for long periods of time and have been epidemiologically defined as HIV resistant [20]. Resistance has not been linked to frequency and duration of condom use, sexually transmitted infections, or differing HIV-1 exposure rates [21] In this study, sex workers who had been followed up for more than 7 years and continue to be HIV-1 uninfected were included. As a control, samples were obtained from an age-matched low risk HIV-1 negative group of non-pregnant women in a monogamous relationship attending a maternal and child health (MCH) clinic To control for confounding effects due to sex work, the outcome was validated against samples from a group of HIV negative sex workers who were recently enrolled in the cohort (New Negatives) and had not met a definitive stage for HESN.

### Sample collection and processing

Whole blood samples were collected in PaxGene blood tubes (Preanalytix, GMBH) and total RNA was extracted in Nairobi following the manufacturer's protocol (Qiagen). Briefly, pelleted nucleic acids were digested with proteinase K then shredded through a spin column to remove protein debris. The resulting supernatant was mixed with ethanol and RNA was selectively bound to a silica-based fibre matrix. Elution of RNA was done with RNASE-free water after washing with saline buffers and digestion with DNAse to remove DNA contamination. Assessment of RNA quality, integrity and purity were done through a Bionalyser 2100 (Agilent Technologies, Palo Alto CA). RNA Samples were considered for further analysis only if they had distinct 28 s and 18 s ribosomal peaks and had been processed or frozen at −70°C within 5 hours after collection.

### Microarray analysis

RNA samples meeting the above criteria were shipped to The Centre For Applied Genomics, Hospitals for Sick Children, Toronto for analysis. 100 ng of RNA was amplified using the Affymetrix two cycle amplification kit (Affymetrix, Santa Clara, CA) incorporating the Ambion MEGAscript T7 amplification procedure. To minimize the challenges posed by abundant globin RNA transcripts, Nugen Whole Blood Solution (Nugen, San Carlos, CA) procedures were incorporated into the Affymetrix protocol. Amplification, labeling, hybridization onto the Affymetrix U133 Plus 2.0 Gene Chip, washing, and scanning were performed as per the manufacturer's protocol.

### Expression analysis

Background subtraction and probe signal summarization was analyzed using the R Bioconductor software. The resulting log2 signal values were retransformed to linear scale and analyzed using the Affymetrix MAS5 package. The program's algorithm output files represented the differences in intensities between the perfect match and mismatch probe sets or a detection of present, marginal or absent calls. Raw image DAT data was processed to CEL files and analyzed using the ArrayAssist software (Stratagene, La Jolla CA). The ratio of the geometric means of the expression analysis of the relevant gene fragments was computed to yield a fold change analysis. Confidence intervals and corrected *p* values were calculated using a Rank Wilcoxson test. Significance was assessed using a 0.5 threshold of the false discovery rate (FDR) which accounts for multiple testing. Only genes with differences between the HESN and controls >1.3 fold and *p* value <0.05 and called as present in at least 50% of the samples tested were considered as differentially expressed. Heatmaps showing differential expression were generated using R and Bioconductor Software [61]. The Database for Annotation, Visualization and Integrated Discovery (DAVID) [62], [63] was used for pathway analysis of the significantly expressed genes.

### Real Time PCR

Selected array results from HESN and controls were validated by quantitative PCR using LightCycler real time PCR (Roche Diagnostics). And to asses that the gene expression profiles may not have been confounded by sex work, blood samples from sex worker women who have been involved in sex work for one year and were HIV negative (New Negatives) were randomly selected and included as additional controls in qRT-PCR confirmation of microarray data.

Samples were analysed with Quantitect SYBR Green RT PCR assay kit as per the manufacturer's protocol (Qiagen). Briefly, 100 ng of each sample were ran in duplicate and normalized to 18 sRNA. Relative expression of each gene was determined from a standard curve of pooled cDNA from human peripheral blood mononuclear cells with total RNA extracted in the same way in a 10 series dilution to form the qRT-PCR standards [64]. *p*-values for significance were analyzed using a student t-test.

#### GeneBank

The microarray data have been deposited at the Genomic Spatial Event (GSE) database under numbers GSE 30155–30240 and GSE 33580.
